# Role of burn severity and posttraumatic stress symptoms in the co-occurrence of itch and neuropathic pain after burns: A longitudinal study

**DOI:** 10.3389/fmed.2022.997183

**Published:** 2022-10-12

**Authors:** N. E. E. Van Loey, A. E. E. de Jong, H. W. C. Hofland, A. I. M. van Laarhoven

**Affiliations:** ^1^Association of Dutch Burn Centres, Maasstad Hospital, Department of Burn Center, Rotterdam, Netherlands; ^2^Department of Clinical Psychology, Utrecht University, Utrecht, Netherlands; ^3^Red Cross Hospital, Burn Center, Beverwijk, Netherlands; ^4^Health, Medical and Neuropsychology Unit, Faculty of Social and Behavioural Sciences, Leiden University, Leiden, Netherlands; ^5^Leiden Institute for Brain and Cognition (LIBC), Leiden University, Leiden, Netherlands

**Keywords:** pruritus, neuropathic pain, scars, posttraumatic stress symptoms, burns

## Abstract

Itch and pain are common after burns. Neuropathic mechanisms may underlie both modalities but remain not well-understood. This study aims to prospectively document neuropathic pain symptoms and to identify potential itch symptom profiles that differ regarding duration and co-occurrence with neuropathic pain which may inform underlying pathophysiological mechanisms and respond to different treatments. Adult burn survivors (*n* = 192) self-reported itch and neuropathic pain at 2 weeks post-discharge, 3, 6, 12, and 18 months post-burn. Based on the presence of itch and pain symptoms over time, participants were allocated to one itch profile: transient itch/pain, chronic itch, or chronic *itch* & *pain*. Profiles were compared on itch *intensity* over time using General Linear Modeling. Age, gender, burn severity, posttraumatic stress (PTS) symptoms and baseline itch intensity were examined as potential predictors of the profiles in a Multi-nominal regression analysis. Neuropathic pain occurred in 54% after discharge which decreased to 24% 18 months later. Itch intensity was highest in the chronic *itch* & *pain* profile. Compared to the transient itch profile, the chronic *itch* & *pain* profile was associated with higher burn severity and more PTS symptoms. Compared to the chronic itch profile, the chronic *itch* & *pain* profile was associated with more PTS symptoms. Findings suggest that biological and psycho-dermatological processes underlie both chronic neuropathic pain and itch processes in burn scars. Further research should elucidate the mechanisms underlying the different itch profiles, with specific focus on skin innervation and psychological factors.

## Introduction

Over the past decade, studies have shown that prevalence rates of pain and itch after the acute phase of burn injury continue to be high. During hospitalization, most patients suffer from pain and itch (1, 2). Although the vast majority of studies shows a subsequent symptom decrease along with scar maturation processes (3, 4), a subgroup develops chronic itch and pain (1, 5) that seems localized within the scars (3, 6). Typically, prevalence rates of itch exceed those of pain [e.g. (7, 8)], indicating itch profiles co-occurring with and without pain. Because pain and itch intensity are highly correlated (9) and share common predictors, severity of both pain and itch may be linked. Examples are e.g., burn severity, particularly related to depth of the wound (1, 3, 10) and posttraumatic stress (PTS) symptoms (10–12). There is convincing evidence for an entangled relationship between chronic pain and PTS symptoms across many patient groups (13). Evidence for a connection between chronic itch and PTS symptoms has also been described (14), but far less studies are currently available compared to pain.

A neuropathic mechanism is assumed to underlie both pain and itch after burns. Neuropathic pain symptoms such as pins and needles, shooting, and burning pain have been described (15, 16), qualifying as spontaneous pain sensations (stimulus-independent) or paresthesia (e.g., burning pain, electric shocks) (17–19). Also itch is assumed neuropathic, particularly after the acute phase when the role of histamine and substance P have abated (20), subscribing that chronic itch seems mostly non-histaminergic (21). Both neuropathic pain and itch can develop after a lesion of the somatosensory system, with involvement of both peripheral and central processes (17, 22). Although peripheral nerve fibers may regenerate after burns, abnormal nerve fiber density in scars has been reported (23). In general, it is assumed that itch is predominantly peripherally activated because, as yet, central sensitization could not be established (6, 24).

Within a neuropathic pathology, also after burns, itch and pain temporally and spatially concur (9). But the underlying neuronal pathways are not fully understood. Current theories, e.g., the labeled line, selectivity, and pattern theory, propose that itch can result from various neuronal pathways, amongst which itch-specialized primary afferent neurons (pruriceptors) and nociceptors are involved (25, 26). Pruriceptors are assumed to transduce itch when being activated by specific molecular markers (e.g., IL-31) (26, 27). Nociceptors may respond to both algogens and pruritogens and are supposed to be differentially activated based on spatial (e.g., focal nociceptive input will produce itch) and temporal aspects of the peripheral input; hence the experienced pain or itch results from the combination of activated fibers (26, 28). Additionally, via inhibitory spinal interneurons, pain signals may inhibit itch transmission. However, in neuropathic itch, it has been put forward that the co-occurrence of itch and pain may result from impaired spinal inhibition, despite current inconclusive evidence (25, 28). Based on the current theories, one may argue that chronic itch and pain after burns may be related and explained by those various mechanisms. Therefore, identifying sensory profiles of itch and pain symptom and biological and psychological differences across the profiles may further elucidate underlying mechanisms.

This 18-months multi-center longitudinal study aims to document neuropathic pain prevalence as well as potential symptom profiles of itch and neuropathic pain that may present after burns, and to explore potential predictors, such as burn severity, age, gender and PTS symptoms potentially related to the symptom profiles.

## Methods

### Patients

This study was part of a larger longitudinal multi-center project examining pain after burn injuries. Previous papers about this project described pain measured with the Brief Pain Inventory (2, 12, 29) but did not focus on itch and neuropathic pain. Patients were included in the study between April 2010 and December 2012 from five burn centers in the Netherlands and in Belgium. Adult patients admitted to the burn centers for >24 h were eligible for inclusion in the study. Exclusion criteria included poor Dutch proficiency, acute or chronic cognitive problems, or when the injury was deliberate. Patients requiring mechanical ventilation were invited to participate as soon as they were able to provide informed consent. During the study period, 340 patients met the inclusion criteria of which 84 declined participation and 40 were missed. A group of 216 patients signed informed consent (64%). They did not differ from the 124 patients not included in the study in terms of age, gender, and affected body area [see also (12)].

### Measures

Neuropathic pain and itch were measured using an adapted version of the self-report Leeds Assessment of Neuropathic Symptoms and Signs (LANSS) Pain Scale (30), a validated screening questionnaire for neuropathic pain (31). The original scale measures the presence (present yes/no) of five symptoms: unpleasant sensations (pricking, tingling, pin-pricks), color differences (motted and looking more red), abnormally sensitive to touch, pain comes suddenly and in bursts (electric shocks), perceived skin temperature (hot or burning). We added to the original version: (1) as part of the item perceived skin temperature, cold sensations were added (original scale only includes hot or burning), (2) a sixth item measuring itch, and (3) in the case that the participant scored “yes,” the intensity of the symptom was scored on a 7-point Likert scale ranging from 0 (not troublesome) to 6 (severely troublesome) which allows to measure the intensity of the symptom, and (4) items were adapted in order to measure pain related to the scars. e.g., “Does the pain feel like a strange and unpleasant sensation *in your scars*?,” and “Does the pain cause *the scar* to look different to normal skin or to that of *scars* that are not painful?.” Unlike the original LANSS, physical assessments of allodynia and altered pin-prick thresholds were not tested in this study because patients self-reported their symptoms. The scale was translated into Dutch by two researchers and back-translated by a native English speaker.

Posttraumatic stress (PTS) symptoms were measured using the Impact of Event Scale-Revised (IES-R) (32). The IES-R measures intrusive, avoidant and hyperarousal symptoms associated with a traumatic event. The original 15 items of the IES (33) and the seven hyperarousal items of the IES-R were used and scored with a 4-point scale (0-1-3-5). The construct validity and reliability of the Dutch version of the IES-R was acceptable (34). Cronbach's alpha was high (0.96). In this study, the 3-month measurement indicative of PTS symptoms rather than acute traumatic stress symptoms was used as a predictor.

Demographic characteristics (i.e., gender and age) and burn severity (i.e., percentage total body surface area (TBSA) burned as well as skin graft procedures) were recorded from the medical file. TBSA burned is the estimated percentage body surface area affected by partial and full-thickness burns.

### Procedure

The study was approved by an ethics committee in the Netherlands (METC Noord-Holland NL27996.094.09) and Belgium (Ghent University B670201112923) and by local institutional review boards of the participating hospitals, and was conducted in accordance with the Helsinki Declaration. Eligible patients were identified by local researchers during admission to the hospital. Oral and written information was provided. Written informed consent was obtained from each patient. Patients completed printed questionnaires in-hospital (e.g., psychological questionnaires), 2 weeks after discharge (T1), 3 months (T2), 6 months (T3), 12 months (T4), and 18 months (T5) after the burn event.

### Data analysis

First, descriptive analyses were performed and patients with complete follow-up were compared with patients who had incomplete follow-up on burn characteristics and demographics using student *t*-tests. Second, itch and neuropathic pain profiles were examined. Two persons (NVL and AvL) independently categorized patients according to the duration of itch and pain [based on literature (3), we used 6 months as cutoff point for chronic itch post-burn which is associated with scar maturation, in contrast to 6 weeks akin the definition of chronic itch resulting from other causes (35)], and potential co-occurrence of neuropathic pain into the following groups: (1) patients reporting itch and/or pain that disappeared after 6 months (*transient itch/pain*); (2) patients reporting itch but never reported pain after 3 months postburn (because we can not exclude that patients may have had small wounds in the postacute phase) (*chronic itch*); (3) patients reporting itch and pain at least 2 out of 5 measurements of which at least once after 6 months (*chronic itch* & *pain*). Beyond the scope of this paper, other profiles included: (4) patients reporting only pain (*chronic pain*); (5) patients reporting no pain or itch (*no pain/itch*). Discrepancies in the categorization of patients were resolved by discussion.

Third, to examine potential differences in the course of itch intensity for the three itch profiles (independent variable), General Linear Modeling (GLM) for repeated measures was conducted with SPSS Statistics for Windows (Version 27.0. Armonk, NY: IBM Corp) with itch intensity over time (T1–T5) as the within-subjects dependent variable and the three itch profiles as independent variable. To investigate potential predictors of the three itch profiles, multi-nominal logistic regression analysis, which uses maximum likelihood estimation to evaluate the probability of categorical membership (of the three itch profiles), was used. Established predictors of both itch and neuropathic pain after burns (age, gender, TBSA burned, surgeries and PTS symptoms) controlling for T1 itch intensity were examined.

## Results

### Patients

Informed consent was provided by 216 patients, but 24 did not complete any of the measurements leaving a final sample size of 192. At discharge (T1), 177 assessments (92%) were available, 166 (86%) at 3 months (T2), 155 (81%) at 6 months (T3), 152 (79%) at 12 months (T4) and 146 (76%) at 18 months (T5). The 146 patients who completed T5 were older [*t*_(213)_ = −4.585, *p* < 0.001], and had higher TBSA burned [*t*_(213)_ = −2.779, *p* = 0.006] and more surgeries [*t*_(213)_ = −2.415, *p* = 0.017] compared to 46 patients lost to follow-up between T2 and T5.

Of the participants, 129 (67%) were male and 63 (33%) were female. Participants were on average 41.56 years old (SD = 15.58). TBSA burned ranged from 1 to 75% (*M* = 9.34, *SD* = 8.85). Ninety participants (46.9%) did not require surgery, 102 (53.1%) needed one or more skin graft procedures. The mean score indexing PTS symptoms was 21.46 (*SD* = 23.89) at 3 months post-burn.

### Itch and pain co-occurrence and itch profiles

**Figure 1:**
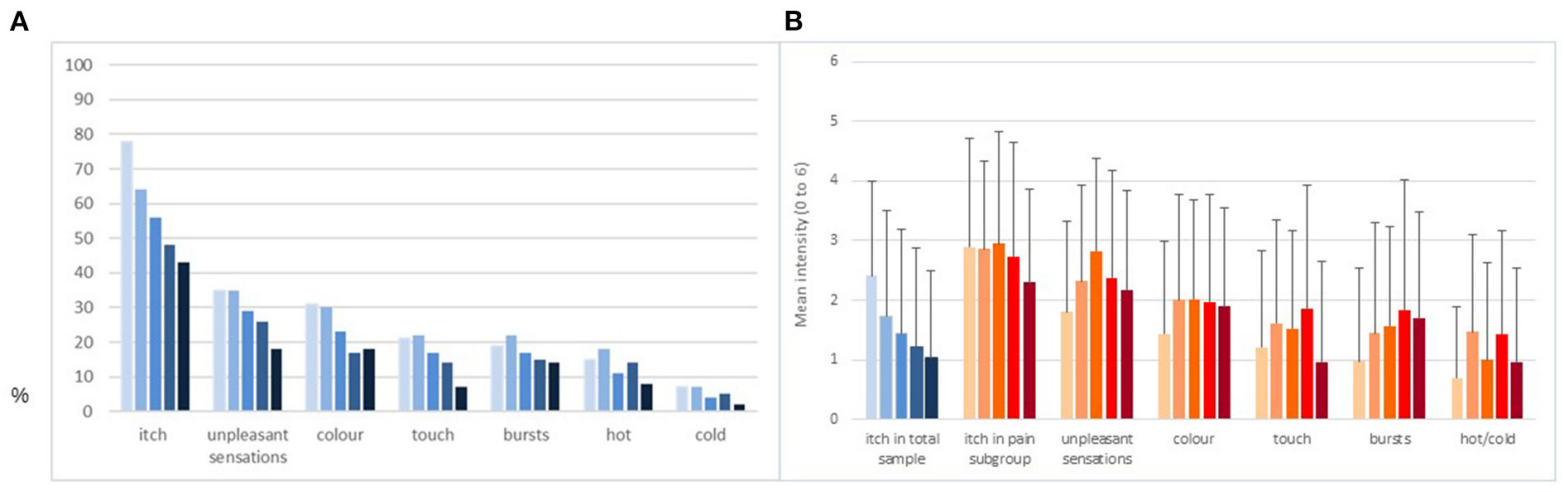
Prevalence rates and observed means of itch intensity and neuropathic pain symptom intensity in complete cohort. **(A)** Percentage of patients indicating the symptom was present. **(B)** Observed means of intensity and standard deviations of the symptoms scored on a 7-point Likert scale. Bars from left to right represent the five time points from 2 weeks post discharge (T1, *n* = 177), 3 (T2, *n* = 166), 6 (T3, *n* = 156), 12 (T4, *n* = 155), and 18 (T5, *n* = 146) months post-burn. Blue bars relate to the total sample. Orange bars relate to the subsample experiencing neuropathic pain symptoms.

The percentage of patients reporting itch decreased over time from 78% (T1) to 43% (T5). For pain, this was 54% (T1) and 24% (T5). Figure 1A presents the prevalence rates of itch and neuropathic pain symptoms (percentage reporting the symptom was present) at the respective time points for the total sample. Whereas itch prevalence rates decreased steadily, pain symptom prevalence remained relatively stable. Figure 1B presents the intensity of itch and pain symptoms measured using a 7-point Likert scale. The blue bars show that itch intensity in the total sample decreased over time, mainly due to the increasing number of patients in which itch disappeared. The orange bars show that itch intensity in the subgroup that also experienced neuropathic pain symptoms was higher on average and more stable. The varying number of patients for every symptom over time can be found in Supplementary Table 1, also presenting additional descriptive details such as mean, standard deviation and median.

Categorization into the different itch profiles was as follows: 51 patients (26.6%) reported *transient itch/pain*, 46 patients (24.0%) reported *chronic itch*, 41 patients (21.4%) reported *chronic itch* & *pain*, 7 patients (3.6%) reported *chronic pain*, 11 patients (5.7%) never reported pain or itch. Twenty-seven patients (14.0%) had no measurements after 6 months, or the symptom pattern could not be attributed to the aforementioned groups (*n* = 9; 4.6%). Table 1 presents the means and standard deviations of the predictor variables for the different profiles. The *chronic itch* & *pain* profile included most patients that needed surgery, had more PTS symptom and comprised more women.

**Table 1:** Descriptive details of predictors for the different itch profiles.

	**Age**	**TBSA burned**	**PTS symptoms**	**Males**	**≥One surgeries**
**Symptom profiles**	**M (SD)**	**M (SD)**	**M (SD)**	***N* (%)**	***N* (%)**
Transient itch/pain (*n* = 51)	43.51 (17.79)	7.65 (5.63)	15.06 (20.79)	40 (78.4)	20 (39.2)
Chronic itch (*n* = 46)	38.63 (16.51)	11.78 (8.52)	20.14 (22.31)	31 (67.4)	29 (63.0)
Chronic itch & pain (*n* = 41)	44.24 (15.44)	12.16 (13.19)	30.32 (26.95)	21 (51.2)	31 (75.6)

M, Means; SD, Standard Deviation; N, number.

### Itch intensity trajectories

**Figure 2:**
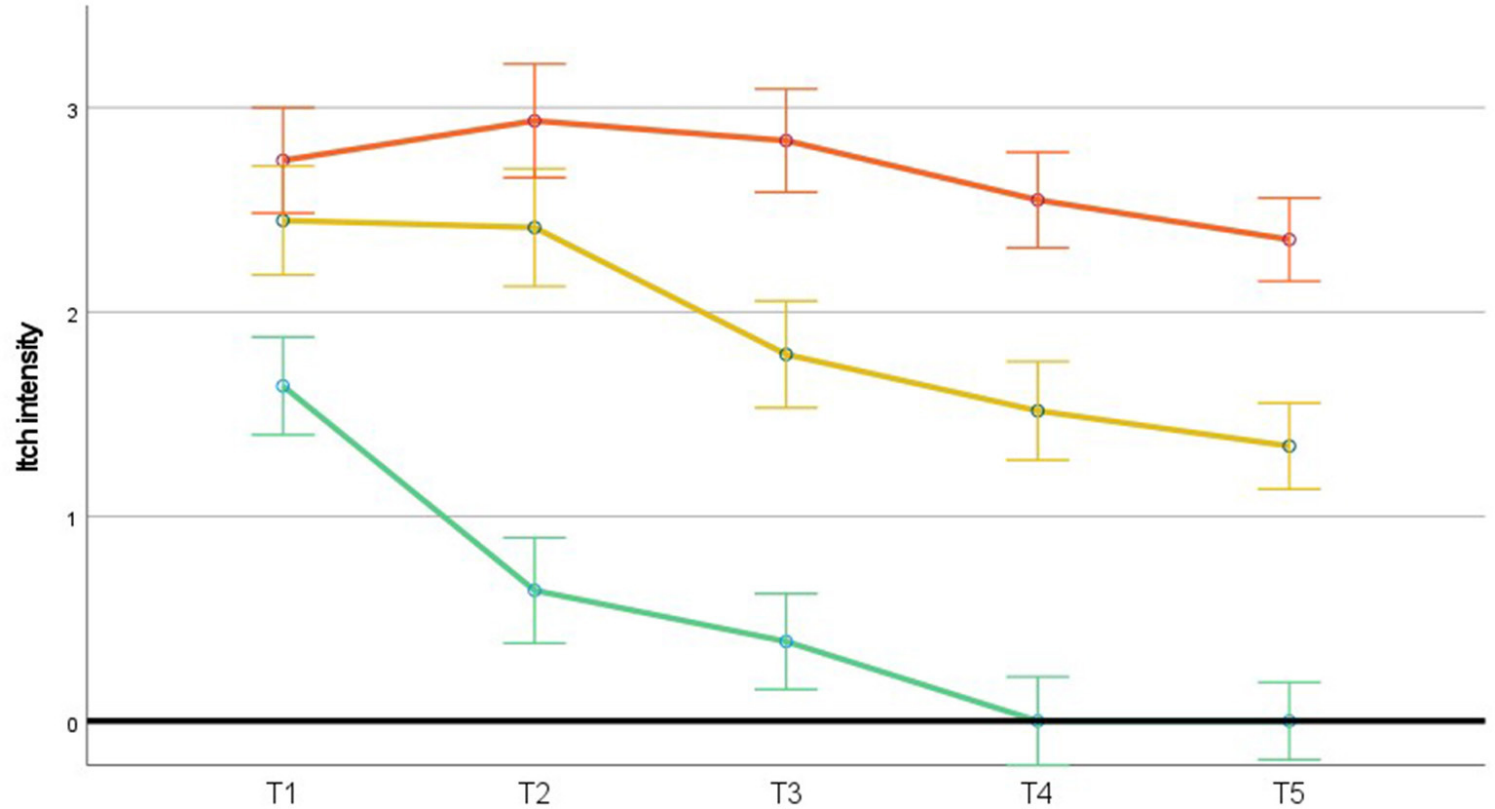
Time course of estimated means of itch intensity (ranging from 0 to 6) in the three itch profiles. *N* = 96 (full cases). Upper line (orange) = chronic *itch* & *pain* (*n* = 31), middle line (yellow) = chronic itch (*n* = 29), lower line (green) = *transient itch/pain* (*n* = 36). Error bars represent 1 standard deviation. T1 = 2 weeks post-discharge, T2 = 3 months post-burn, T3 = 6 months post-burn, T4 = 12 months post-burn and T5 = 18 months post-burn.

GLM was used to study possible differences in itch intensity over time across the three itch profiles. Figure 2 shows that itch intensity was highest in the *chronic itch* & *pain* profile. The main effect of the profiles was significant, [*F*(2, 93) = 44.80, *p* < 0.001], as was the main linear effect of time, [*F*(1, 93) = 36.925, *p* < 0.001]. This suggests that both the three itch profiles and time explain variation in itch intensity and therefore are relevant to consider. The interaction of these two factors (i.e., the itch profiles and time) was also significant, [*F*(2, 93) = 3.409, *p* = 0.037]. This indicates that the profiles show different patterns of itch intensity over time. Figure 2 shows that patients reporting *transient itch/pain* showed, unsurprisingly, an early steep decline in itch intensity ultimately resulting in complete itch alleviation. Of more interest is the difference between the two chronic profiles, where in both profiles, itch intensity slightly decreased, but remained substantial, with higher itch intensity in the chronic *itch* & *pain* profile than in the chronic itch profile.

### Predictors of itch profiles

Using multi-nominal logistic regression analysis, we tested whether the three itch profiles were associated with differences regarding gender, burn severity (TBSA burned and needing surgery) age, PTS symptoms and itch intensity measured post-discharge. The fit between the model containing only the intercept and data improved with the inclusion of the predictor variables [χ^2^(12, n = 112) = 43.09, *p* < 0.001, Nagelkerke *R*^2^ = 0.279]. This indicates that inclusion of the predictors is meaningful and explains variance across the profiles. In the upper part of Table 2A the *transient itch/pain* profile was the reference group which means that the outcomes of the two chronic itch profiles were compared to the *transient itch/pain* profile. The results revealed that compared to *transient itch/pain* profile, younger patients (*p* = 0.045) and those with a larger TBSA burned (*p* = 0.034), and needing surgery (*p* = 0.060) were more likely to be assigned to *the chronic itch* profile. Needing surgery (*p* = 0.004) and higher levels of PTS symptoms (*p* = 0.024) increased the likelihood to be assigned to the *chronic itch* & *pain* profile. In the lower part of Table 2B, the *chronic itch* & *pain* profile was the reference category which allowed to investigate differences across the two chronic profiles. The results showed one statistically significant difference: PTS symptom levels were higher in the *chronic itch* & *pain* profile (*p* = 0.019).

**Table 2:** Burn characteristics and demographics tested with multi-nominal regression analysis to predict classification into one of the three itch profiles.

	**B**	**SE**	**Wald**	**df**	**Sig**	**Exp(B)**	**95% CI**	
**Reference category is transient itch/pain**
**Chronic itch**							**Upper**	**Lower**
Intercept	0.992	1.151	0.744	1	0.388			
Age	−0.036	0.018	4.028	1	0.045	0.965	0.931	0.999
Male (=0)	−0.607	0.600	1.025	1	0.311	0.545	0.168	1.765
PTS symptoms	0.001	0.013	0.002	1	0.965	1.001	0.975	1.027
TBSA burned	0.085	0.040	4.512	1	0.034	1.088	1.007	1.176
No surgery (=0)	−1.003	0.534	3.537	1	0.060	0.367	0.129	1.043
Itch T1	0.217	0.200	1.175	1	0.278	1.242	0.839	1.837
**Chronic itch** & *pain*
Intercept	0.233	1.246	0.035	1	0.852			
Age	−0.016	0.019	0.672	1	0.412	0.984	0.948	1.022
Male (=0)	−1.065	0.610	3.046	1	0.081	0.345	0.104	1.140
PTS symptoms	0.029	0.013	5.060	1	0.024	1.030	1.004	1.056
TBSA burned	0.052	0.043	1.421	1	0.233	1.053	0.967	1.146
No surgery (=0)	−1.808	0.622	8.461	1	0.004	0.164	0.048	0.554
Itch T1	0.210	0.211	0.987	1	0.320	1.233	0.815	1.866
**Reference category is chronic itch** & *pain*
**Chronic itch**							**Upper**	**Lower**
Intercept	0.759	1.125	0.455	1	0.500			
Age	−0.020	0.018	1.233	1	0.267	0.980	0.946	1.015
Male (=0)	0.458	0.554	0.684	1	0.408	1.581	0.534	4.681
PTS symptoms	−0.029	0.012	5.459	1	0.019	0.972	0.949	0.995
TBSA burned	0.033	0.033	1.014	1	0.314	1.034	0.969	1.102
No surgery (=0)	0.805	0.614	1.720	1	0.190	2.237	0.672	7.449
Itch T1	0.007	0.184	0.001	1	0.971	1.007	0.702	1.445

B represents the regression coefficient. SE, standard error; df, degrees of freedom; Exp (B), exponent B; CI, confidence interval.

## Discussion

This study prospectively documents itch and neuropathic pain symptom development and investigated itch profiles regarding duration and co-occurrence with neuropathic pain in adult burn survivors. Neuropathic pain symptoms were reported by 54% of the participants at 2 weeks post-discharge which declined to 24% at 18 months postburn. For itch, prevalence rates were 78% at 2 weeks postdischarge and 43% at 18 months postburn. Itch intensity was most severe in the *chronic itch* & *pain* profile. Compared to the *transient itch/pain* profile, both chronic profiles were associated with more severe burns, and the *chronic itch* & *pain* profile was associated with more PTS symptoms.

Patients' symptom profiles differ regarding co-occurrence with neuropathic pain, including *transient itch/pain, chronic itch* and *chronic itch* & *pain*. Itch intensity in the *transient itch/pain* profile showed a rapid decrease and the patients had less severe burns. This corroborates earlier findings that partial thickness burns more likely produce temporal itch (3) and may be predominantly histaminergic evoked in the early phase of wound healing streching out to the early remodeling phase in which antihistamines provide relief in a subgroup of patients (20).

Significantly higher itch intensities were perceived by the patients within the *chronic itch* & *pain* profile compared to those in the *chronic itch* profile. Provisional support for an association of mixed sensations and symptom severity may come from a study in which patients reporting both neuropathic pain and itch more likey needed both gabapentin and pregabalin compared to patients reporting itch only who received gabapentin to achieve symptom relief (36). Both chronic profiles were associated with more severe burns than the *transient itch/pain* profile. Particularly wounds that needed surgery, i.e., full thickness burns, may have affected skin innervation patterns. The newly regenerating nerve branches may evoke itch and/or pain due to spatial arrangement and spontaneous activity in regenerating sprouts and/or local inflammation (28). When only few epidermal nociceptors are focally activated and many are not, those may produce itch which is described as a “mismatch signal” (25, 37). Possibly, itch-specific pathways are involved in which mediators such as IL-31, pruriceptive neurons, and spinal neurons expressing gastrin-releasing peptide (GRP) play a role, although the latter may also apply to nociceptors (26). Increased levels of IL-31 have been identified in hypertrophic burn scars (38) but the involvement of GRP has not been investigated to our knowledge. What remains unclear is why the co-occurrence of pain and itch produces higher itch intensity. Although speculative, reduced descending inhibition may play a role. Future research may focus on different neuronal pathways in burn scars that may explain variation in itch intensity as well as temporal and spatial co-occurrence of itch and pain.

Higher PTS symptom levels were particularly associated with the *chronic itch* & *pain* profile. This is in line with studies showing a link between PTS symptoms and higher itch intensity (10, 39). Possibly, PTS symptoms affect central processing, potentially decreasing the threshold for pain, and perhaps also for itch. As shown in a study using electroencephalography (EEG) oscillatory activity, itch and pain seem processed differently in burn survivors with PTS symptoms compared to those without PTS symptoms (24). We could speculate that PTS symptoms may influence top-down sensory predictions, which play an important role in symptom perception (40). Due to the repeated peripheral somatosensory input (bottom-up), the brain has learnt to predict upcoming somatosensory sensations. This can result in the actual neurobiological perception of symptoms in the brain becoming aligned with the prediction via active interoceptive inference. Especially when sensory input is imprecise and in case of chronic symptoms, predictive processes are supposed to significantly modulate perception (40, 41). In this light, the threat resulting from a traumatic burn event and associated pain may form strong perceptual priors with a high probability, modifying later sensory perceptions, including pain and itch, corroborating that PTS symptoms amplify predictive coding processes (42). Another explanation may relate to increased production of peripheral inflammatory mediators. Elevated corticosteroids and alterations in cytokines related to psychological stress have been associated with slower wound healing (43) and an association between PTS symptoms and lower oxytocin levels in burn wounds have been found (44). This suggest that PTS symptoms can also exert an effect at skin-level through increased production of excitatory skin mediators, one of the mechanisms explaining neuropathic itch (28) and calls for more attention to identify and treat PTS symptoms.

A small effect of younger age was found associated with the categorization to the chronic itch profile, which corroborates earlier findings (1, 45) of which the authors explained the effect of age by neurological and vascular aging of the skin.

The neuropathic pain symptoms prevalence rate of 54% 2 weeks post-discharge was high compared to a study that reported 28% pain at 6 weeks post-burn (9), but 6 to 18 month prevalence rates were within the same range, be it 24% in the current study vs. 21% in the study of Mauck et al. However, it is substantially higher than the 6% prevalence rate (113/1,880 patients) reported in a retrospective chart review study (46). Likely, the prospective and systematic examination of pain symptoms explains the higher prevalence rates. In line with other studies, unpleasant sensations such as pin-pricks was the most frequently reported neuropathic pain symptom, e.g. (15). But also other symptoms such as bursts, sensitive touch, and burning pain were reported which overall indicates that pain symptoms in scars are of neuropathic origin.

This study has clinical and research implications. First, the itch profiles may inform clinical practice and future research into treatments. It is recommended to screen patients for sensory symptoms, specifically focused at the co-occurrence of itch and neuropathic pain, and other risk factors to tailor prescription of for instance gabapentin or pregabalin in an earlier stage (36). Second, more clinical attention to detect and treat PTS symptoms in an early phase is recommended as it may also improve pain and itch outcomes. Third, results call for further exploration of the involvement of different neuronal pathways and contribution of central sensitization processes in the various itch profiles, that may pave the way to conduct targeted medication clinical trials. For example, psychophysically assessing itch and pain modulation may predict course of symptoms and therapeutic (e.g., post-operative) outcomes for both chronic itch and pain (47, 48).

This study also has limitations. First, although the literature is positive about using self-report questionnaires to assess spontaneous pain-related sensations (31), allodynia, and loss of sensory function were not measured because clinical examination is required (49). Loss of sensory function or numbness has been documented in burn scars, indicating its relevance (50). Clinical assessments that document stimulus-evoked sensory sensations and temporal summation may inform underlying nerve damage and consequently, therapy (49). Second, the LANSS was modified, including the addition of a 7-point Likert scale, and warrants further validation along with clinical tests to establish its reliability and validity in burn scars. Third, the sample size of the different profiles was small which limits statistical power. Consequently, replication research is warranted. Additionally, the small sample size was deemed too small to use more sophisticated statistical analyses to explore latent classes (read: itch profiles), which could replace the classification of participants based on the duration of the complaints and itch-pain co-occurrence.

In conclusion, the current study shows that the co-occurrence of chronic itch and chronic neuropathic pain is associated with higher itch intensity compared to chronic itch only. This suggests different underlying mechanisms, perhaps related to different neuronal pathways or differences in modulation systems, but this should be considered as hypothesis generating. The role of PTS symptoms may point to altered central processing, which may be another pathway explaining higher itch intensity. Future research focussing on peripheral and central processing of itch from a bio-psychological perspective is warranted. This may ultimately inform pathophysiological and pharmacological mechanisms in future studies—and hence lead to better treatment and improved quality of life of individuals after burn injury.

## Data availability statement

The raw data supporting the conclusions of this article will be made available by the authors, upon reasonable request.

## Ethics statement

The studies involving human participants were reviewed and approved by METC Noord-Holland, Netherlands & Ghent University, Belgium. The patients/participants provided their written informed consent to participate in this study.

## Author contributions

Conception and/or design of the study: NVL, AdJ, and HH. Interpretation of the data and drafted the work: NVL and AvL. Revision the paper: AdJ and HH. All authors approved the final version and agree to be accountable for all aspects of the work.

## Funding

Funding was provided by Fonds NutsOhra Grant Numbers 0901-057 and 1101-035.

## Conflict of interest

The authors declare that the research was conducted in the absence of any commercial or financial relationships that could be construed as a potential conflict of interest.

## Publisher's note

All claims expressed in this article are solely those of the authors and do not necessarily represent those of their affiliated organizations, or those of the publisher, the editors and the reviewers. Any product that may be evaluated in this article, or claim that may be made by its manufacturer, is not guaranteed or endorsed by the publisher.
