# Supplementary material for: Role of burn severity and posttraumatic stress symptoms in the co-occurrence of itch and neuropathic pain after burns: A longitudinal study
Source: Front Med (Lausanne). 2022 Oct 12;9:997183. doi: 10.3389/fmed.2022.997183 (PMC9596796; doi:10.3389/fmed.2022.997183)
Supplement: Supplementary file 1 [file Table_1.pdf]

Supplementary Table 1 Prevalence, Mean, Standard Deviation and Median of pain(symptoms) and itch

		Pain	Neuropathic pain symptoms					Itch
		Yes/no	Unpleasant sensations	Color	Touch	Bursts	Hot/cold	Yes/no
T1 (n = 177)								
	Prevalence	n=95 (54%)	n=62	n=54	n=37	n=33	n=26/12	n=137 (78%)
	Mean		1.81	1.43	1.21	.98	.70	2.41
	Mdn		2.00	1.00	.00	.00	.00	2.00
	SD		1.52	1.56	1.62	1.55	1.19	1.58
	Pain itch overlap	n=81 (86%)						N=81 (59%)
T2 (n = 166)								
	Prevalence	n=71 (43%)	n=58	n=50	n=36	n=36	n=30/12	n=106 (64%)
	Mean		2.32	2.01	1.61	1.45	1.47	1.73
	Mdn		2.00	2.00	1.00	.00	1.00	1.00
	SD		1.61	1.77	1.73	1.85	1.62	1.78
	Pain itch overlap	n=60 (85%)						n=60 (57%)
T3 (n = 156)								
	Prevalence	n=52 (33%)	n=45	n=36	n=27	n=27	n=17/7	n=87 (56%)
	Mean		2.83	2.02	1.52	1.57	1.00	1.45
	Mdn		3.00	2.00	1.00	1.00	.00	1.00
	SD		1.54	1.67	1.65	1.66	1.62	1.74
	Pain itch overlap	n=42 (82%)						n=42 (48%)
T4 (n=155)								
	Prevalence	n=36 (24%)	n=31	n=26	n=21	n=22	n=21/7	n=73 (48%)
	Mean		2.38	1.97	1.86	1.84	1.43	1.21
	Mdn		2.00	2.00	1.00	1.00	1.00	.00
	SD		1.80	1.81	2.06	2.18	1.74	1.67
	Pain itch overlap	n=29 (81%)						n=29 (40%)
T5 (n = 146)								
	Prevalence	n=35 (24%)	n=26	n=26	n=10	n=21	n=11/3	n=63 (43%)
	Mean		2.17	1.89	.97	1.69	.97	1.03
	Mdn		2.00	2.00	.00	1.00	.00	.00

	SD		1.67	1.66	1.69	1.80	1.58	1.47
	Pain itch overlap	n=26 (74%)						n=26 (41%)

Note. SD = standard deviation; Mdn = Median, n = number
